# Loss of Smyhc1 or Hsp90α1 Function Results in Different Effects on Myofibril Organization in Skeletal Muscles of Zebrafish Embryos

**DOI:** 10.1371/journal.pone.0008416

**Published:** 2010-01-01

**Authors:** Marta Codina, Junling Li, Joaquim Gutiérrez, Joseph P. Y. Kao, Shao Jun Du

**Affiliations:** 1 Center of Marine Biotechnology, Biotechnology Institute, University of Maryland, Baltimore, Maryland, United States of America; 2 Department of Physiology, University of Barcelona, Barcelona, Spain; 3 Medical Biotechnology Center, Biotechnology Institute, University of Maryland, Baltimore, Maryland, United States of America; 4 Center for Biomedical Engineering and Technology, and Department of Physiology, University of Maryland, Baltimore, Maryland, United States of America; 5 Interdisciplinary Training Program in Muscle Biology, University of Maryland School of Medicine, Baltimore, Maryland, United States of America; Louisiana State University, United States of America

## Abstract

**Background:**

Myofibrillogenesis requires the correct folding and assembly of sarcomeric proteins into highly organized sarcomeres. Heat shock protein 90α1 (Hsp90α1) has been implicated as a myosin chaperone that plays a key role in myofibrillogenesis. Knockdown or mutation of *hsp90α1* resulted in complete disorganization of thick and thin filaments and M- and Z-line structures. It is not clear whether the disorganization of these sarcomeric structures is due to a direct effect from loss of Hsp90α1 function or indirectly through the disorganization of myosin thick filaments.

**Methodology/Principal Findings:**

In this study, we carried out a loss-of-function analysis of myosin thick filaments via gene-specific knockdown or using a myosin ATPase inhibitor BTS (N-benzyl-p-toluene sulphonamide) in zebrafish embryos. We demonstrated that knockdown of myosin heavy chain 1 (*myhc1*) resulted in sarcomeric defects in the thick and thin filaments and defective alignment of Z-lines. Similarly, treating zebrafish embryos with BTS disrupted thick and thin filament organization, with little effect on the M- and Z-lines. In contrast, loss of Hsp90α1 function completely disrupted all sarcomeric structures including both thick and thin filaments as well as the M- and Z-lines.

**Conclusion/Significance:**

Together, these studies indicate that the *hsp90α1* mutant phenotype is not simply due to disruption of myosin folding and assembly, suggesting that Hsp90α1 may play a role in the assembly and organization of other sarcomeric structures.

## Introduction

Muscle fibers are composed of myofibrils, one of the most highly ordered macromolecular assemblies in cells. Each myofibril is made up of repetitive organized structures called sarcomeres, the basic contractile unit in skeletal and cardiac muscles. The sarcomere is composed of myosin (thick) and actin (thin) filaments. Myosin and actin proteins are assembled to form the highly organized thick and thin filaments with the help of titin, nebulin, and other structural proteins in the Z-disks and M-bands [1], [2], [3], [4], [5], [6]. The regulatory mechanisms that lead to the formation of this highly organized sarcomeric structure have been extensively investigated in cell culture *in vitro*; however, the regulatory mechanism is not yet completely understood during muscle development *in vivo* [7], [8], [9].

Genetic studies and biochemical analyses have shown that chaperone-mediated myosin folding and assembly is an integral part of myofibrillogenesis during muscle development. Mutations of *Caenorhabditis elegans UNC-45*, a myosin chaperone, result in paralyzed animals with severe myofibril defects in body wall muscles [10], [11], [12], [13], [14]. Recent studies demonstrated that heat shock protein 90α1 (Hsp90α1) which binds to UNC-45 and forms a complex with newly synthesized myosin proteins also plays a vital role in myosin folding and myofibril assembly [10], [15], [16], [17], [18]. Hsp90α1 is specifically expressed in developing somites and skeletal muscles of zebrafish embryos [15], [19]. Knockdown or mutation of *hsp90α1* resulted in paralyzed zebrafish embryos with defective skeletal muscle contraction [15], [16], [17]. Assembly of thick and thin filaments, as well as the M- and Z-lines, was completely disrupted in skeletal muscles of *hsp90α1* knockdown or mutant zebrafish embryos [15], [17]. It is not clear whether the myofibril defects in other sarcomeric structures of *hsp90α1* knockdown embryos were caused directly from *hsp90α1* knockdown or indirectly through the effect of disorganization of myosin thick filaments.

In this study, we directly knocked down myosin heavy chain expression in slow muscles of zebrafish embryos and compared the muscle phenotypes with that of the *hsp90α1* mutant (*slo^tu44c^*) that carries a nonsense mutation in the C-terminal region. We demonstrated that knockdown of *smyhc1* had a different effect than *hsp90α1* mutation on sarcomere assembly. Unlike *hsp90α1* mutation which completely disrupted all sarcomeric structures, knockdown of myosin resulted in a restricted sarcomeric defect in thick and thin filaments. This was further confirmed by treating zebrafish embryos with BTS (N-benzyl-p-toluene sulphonamide), a specific inhibitor for myosin ATPase and myosin-actin interaction in zebrafish embryos. Together, these studies demonstrated loss of myosin function resulted in a different effect than *hsp90α1* mutation on myofibril organization, suggesting the *hsp90α1* mutant phenotype is not simply due to disruption of myosin folding and assembly.

## Results

### 1. Knockdown of smyhc1 Expression Resulted in Paralyzed Zebrafish Embryos with Defective Thick Filament Organization in Slow Muscles

**Figure 1:**
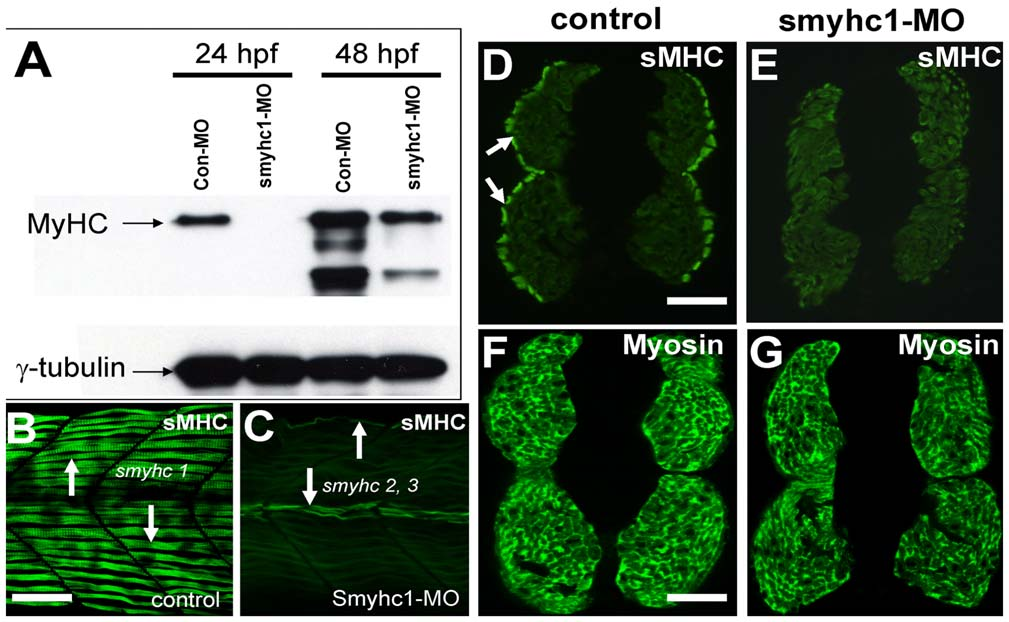
Knockdown of *smyhc1* expression by *smyhc1* ATG-MO. A. Western blot analysis shows the effect of *smyhc1*-MO on the expression of the myosin heavy chain in slow muscles (F59) at 24 and 48 hpf. Anti-γ-tubulin was used as a loading control. B, C. Anti-MyHC antibody (F59) staining shows myosin expression in trunk slow muscles of control (B), or *smyhc1* knockdown (C) embryos at 48 hpf. Myosin expression was significantly knocked down in slow muscles. However, myosin expression could be detected in myofibers in the dorsal and myoseptum region of the myotome (arrows) that express *smyhc2 and smyhc3*. D, E. F59 antibody staining on cross-sections shows MyHC expression in slow muscles (arrows) of control (D) or *smyhc1*-ATG-MO injected embryos (E) at 48 hpf. F, G. MF20 antibody staining shows MyHC expression in fast muscles of control (F) or *smyhc1*-ATG-MO (G) injected embryos at 48 hpf. Scale bars = 25 µm in B; 75 µm in D and F.

Zebrafish embryonic muscles can be divided into two major types, slow and fast, based on the expression of myosin heavy chain (MyHC). Smyhc1 represents the first and primary MyHC expressed in slow muscles of zebrafish embryos that can be labeled specifically with F59 monoclonal antibody [20], [21], [22]. In addition, two other *myhc* genes, *smyhc2* and *smyhc3*, are also expressed in a small subset of zebrafish slow muscles at the dorsal, ventral and myoseptum regions of the myotome in late stage embryos [20], [21], [23]. To determine the myosin knockdown phenotype and compare it with that of *hsp90α1* mutation, we knocked down *smyhc1* expression using a *smyhc1*-specific translational morpholino (ATG-MO) in zebrafish embryos (Fig. 1). The ATG-MO was targeted to the flanking sequence of the *smyhc1* ATG start codon. It shares 50–70% identity with the corresponding sequences in zebrafish *smyhc2*, *smyhc3*, *fmyhc2* and *fmyhc4*.

Western blot analyses showed a significant reduction of Smyhc1 protein levels in *smyhc1* ATG-MO injected zebrafish embryos (Fig. 1A). Immunostaining of whole mount embryos confirmed that myosin expression was missing or greatly reduced in slow muscles of the knockdown zebrafish embryos (Fig. 1C, E). However, expression of other MyHCs in fast muscles was not affected (Fig. 1G). Moreover, myosin expression in a subset of slow muscles in the dorsal and myoseptum regions of the myotome that express smyhc2 and smyhc3 also appeared normal (Fig. 1C). Together, these data indicate that the *smyhc1* ATG-MO was specific in knocking down the expression of *smyhc1* in slow muscles of zebrafish embryos.

**Figure 2:**
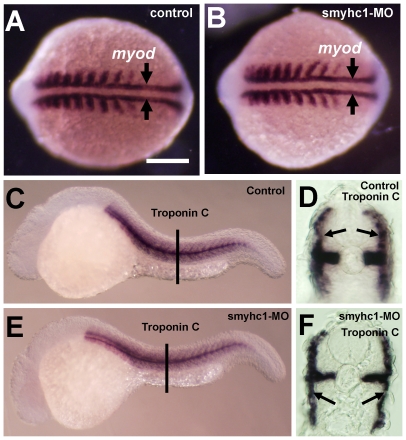
Effects of *smyhc1* knockdown on muscle development in zebrafish embryos. A and B. *In situ* hybridization shows *myod* expression in control (A) or *smyhc1*-ATG-MO (B) injected embryos at 14 hpf. Adaxial cells that give rise to slow muscles are indicated by arrows. C–F. *In situ* hybridization shows slow-specific *troponin C* expression in control (C, D) or *smyhc1*-ATG-MO (E, F) injected embryos at 24 hpf. D and F are cross sections of C and E, respectively. Arrows in D and F indicate slow muscles.

To determine whether knockdown of *smyhc1* affects slow muscle development, we analyzed *myod* expression by *in situ* hybridization. Compared with controls (Fig. 2A), a similar pattern of *myod* expression was observed in *smyhc1* knockdown embryos (Fig. 2B). Two rows of *myod*-expressing adaxial cells that give rise to slow muscles were clearly detected in the *smyhc1* knockdown embryos (Fig. 2B), confirming that knockdown of *smyhc1* did not alter the initial formation of slow muscle precursors in zebrafish embryos. To determine whether slow muscle differentiation was affected in *smyhc1* knockdown embryos, we analyzed the expression of slow muscle-specific *troponin C* in the knockdown embryos. The results showed that *smyhc1* knockdown did not affect the expression of slow-specific *troponin C* in zebrafish embryos at 24 hpf (Fig. 2E, F). Together, these data confirmed that knockdown of *smyhc1* did not affect the formation of slow muscles.

**Figure 3:**
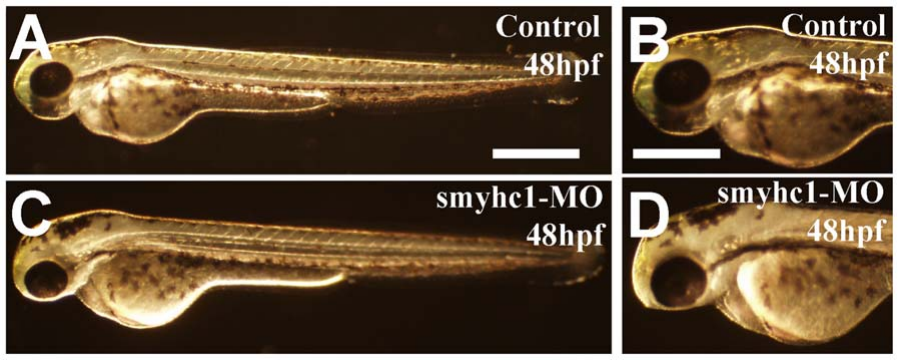
Morphology of control and *smyhc1* knockdown embryos at 48 hpf. Morphological comparison of control (C, D) *or smyhc1*-ATG-MO injected (E, F) embryos at 48 hpf. Scale bars = 30µm in A, 100 µm in C and D.

To determine whether knockdown of *smyhc1* affected muscle function, the *smyhc1* ATG-MO injected embryos were examined morphologically following the *smyhc1* ATG-MO injection. Although the injected embryos appeared morphologically normal compared with control (Fig. 3), *smyhc1* knockdown embryos were paralyzed at 24 hpf, a phenotype very similar to the zebrafish *slo^tu44c^* mutant carrying a nonsense mutation in the *hsp90α1* gene [17].

**Figure 4:**
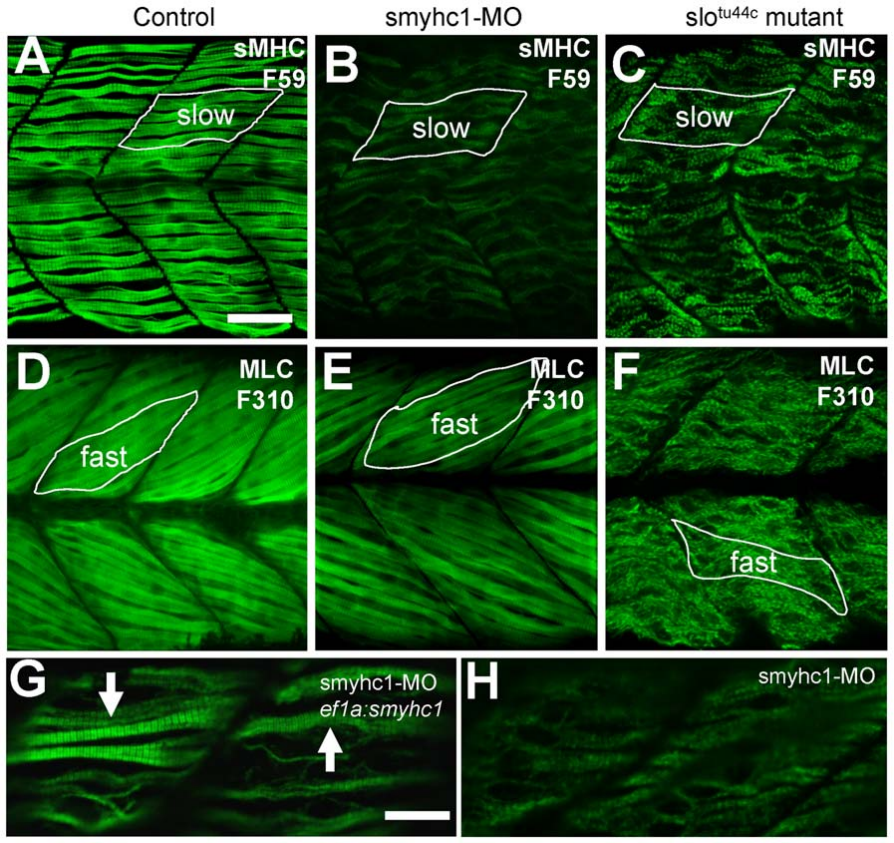
Effects of *smyhc1* knockdown or *hsp90α1* mutation on myosin thick filament organization in skeletal muscles of zebrafish embryos. A–C. Anti-MyHC antibody (F59) staining shows the organization of thick filaments in trunk slow muscles of control (A), *smyhc1* knockdown (B), or *slo^tu44c^* mutant (C) embryos at 48 hpf. D–F. Anti-MLC antibody (F310) staining shows the organization of thick filaments in trunk fast muscles of control (D), *smyhc1* knockdown (E), or *slo^tu44c^* mutant (F) embryos at 72 hpf. Note, fast fibers project with a 30 degree angle with respect to the axial structure, whereas slow fibers project in parallel to the axial structure. G, H. Anti-MyHC antibody (F59) staining shows the rescue of thick filaments in smyhc1 knockdown zebrafish embryos co-injected with *ef1a:smyhc1* DNA construct (G), or ATG-MO alone (H). Scale bar = 25 µm in A, 10 µm in G.

To determine whether knockdown of *smyhc1* expression resulted in defective thick filament organization, we examined the organization of myosin thick filaments in *smyhc1* knockdown embryos by immunostaining with the F59 and F310 antibodies that label myosin in slow and fast muscles, respectively. Compared with *slo^tu44c^* mutant zebrafish embryos (Fig. 4C), knockdown of *smyhc1* expression resulted in no organized thick filaments in slow muscles of zebrafish embryos (Fig. 4B). This was expected considering the *smyhc1* is specifically expressed in slow muscles. However, knockdown of *smyhc1* had no effect on thick filament organization in fast muscles (Fig. 4E). This was in contrast to *slo^tu44c^* mutant that has muscle defects in both slow and fast muscles (Fig. 4F). Together, these data indicate that Smyhc1 is required for thick filament assembly in slow muscles.

To confirm the specificity of phenotype, we performed a rescue experiment by co-injecting a *smyhc1* expression DNA construct with the *smyhc1* ATG-MO into zebrafish embryos. The ATG-MO was not able to knock down the expression of the DNA construct because its 5′ UTR sequence targeted by the ATG-MO has been replaced with a 5′ UTR sequence from the b-globin gene. The results showed that transient expression of the *smyhc1* DNA construct could rescue the thick filament defect (Fig. 4G). The rescued myofibers appeared in a mosaic fashion, consistent with the typical mosaic pattern of gene expression from DNA injection.

### 2. Knockdown of smyhc1 Expression Disrupted Organization of Thin Filaments in Slow Muscles

**Figure 5:**
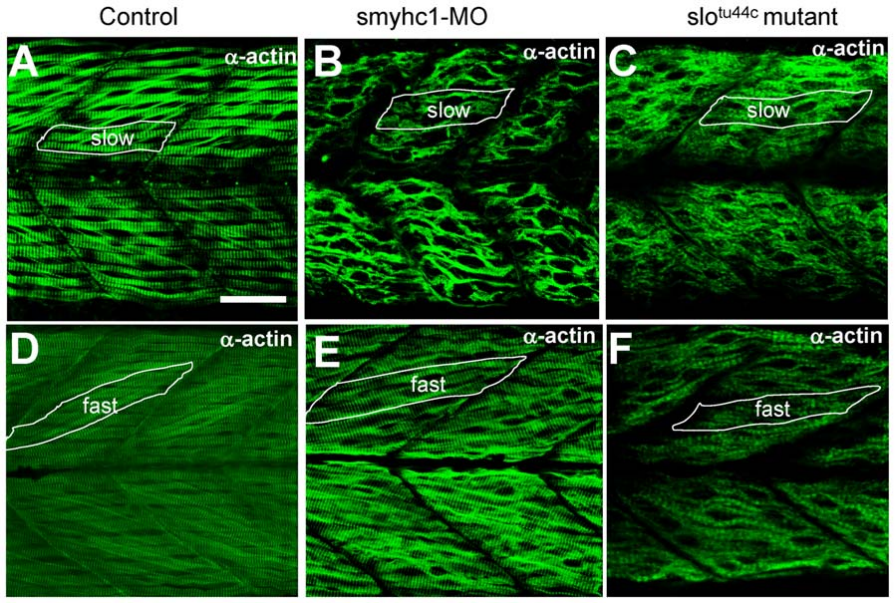
Knockdown of *smyhc1* expression or *hsp90α1* mutation resulted in defective thin filament organization in skeletal muscles of zebrafish embryos. A–C. Anti-α−actin antibody staining shows the organization of thin filaments in slow muscles of control (A), *smyhc1* knockdown (B), or *slo^tu44c^* mutant (C) embryos at 48 hpf. D, F. Anti-α−actin antibody staining shows the organization of thin filaments in fast muscles of control (D), *smyhc1* knockdown (E), or *slo^tu44c^* mutant (F) embryos at 72 hpf. Scale bar = 25 µm in A.

During myofibrillogenesis, actin thin filaments align around myosin thick filaments in a hexagonal arrangement to form the highly ordered sarcomeric structure. It has been suggested that interaction with myosin is critical for α-actin thin filament organization. To test whether thin filament organization was affected in *smyhc1* knockdown embryos, we examined thin filaments in *smyhc1*ATG-MO injected embryos by immunostaining with anti-α-actin antibody. Compared with the control-MO injected embryos (Fig. 5A), *smyhc1* knockdown embryos showed disorganized thin filaments in slow muscles (Fig. 5B). The thin filament defects were very similar to that observed in *hsp90α1* mutant zebrafish embryos (Fig. 5C). As expected, the thin filament defects were specifically restricted to slow muscles in *smyhc1* knockdown embryos. Thin filaments in fast muscles appeared normal in *smyhc1* knockdown embryos (Fig. 5E). This differs from the *slo^tu44c^* mutant, which exhibited thin filament defects in both slow and fast muscles (Fig. 5F). Together, these data suggest that disruption of myosin thick filaments could result in defective organization of thin filaments in muscle cells.

### 3. Inhibition of Myosin Function by BTS Resulted in Defective Thick and Thin Filaments in Skeletal Muscles of Zebrafish Embryos

**Figure 6:**
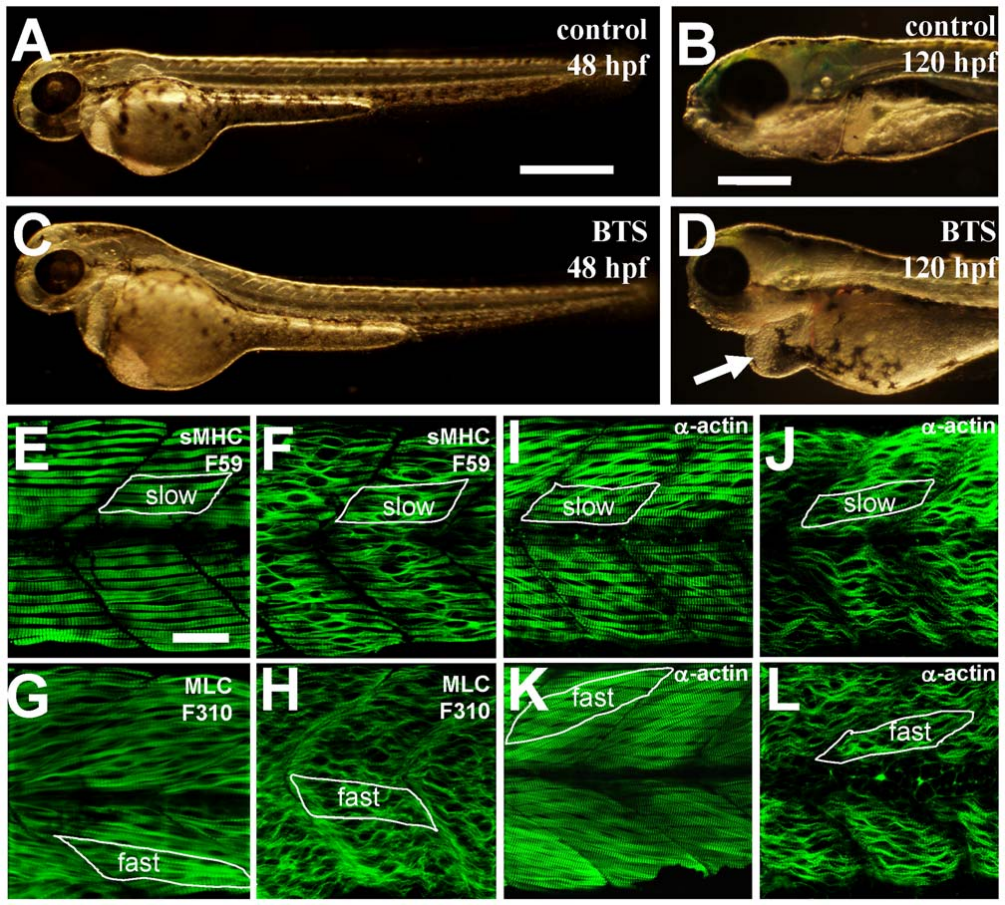
BTS inhibits skeletal muscle contraction and suppresses thick and thin filament assembly in skeletal muscles of zebrafish embryos. A–D. Morphological comparison of control (A, B) or BTS-treated (C, D) embryos at 48 hpf (A, C) and 120 hpf (B, D). Compared with control (B), BTS-treated embryos (D) showed a clear edema (indicated by the arrow) at 120 hpf. E and F. Anti-MyHC antibody (F59) staining shows the organization of thick filaments in slow muscles of control (E) or BTS-treated (F) embryos at 60 hpf. G and H. Anti-MLC antibody (F310) staining shows the organization of thick filaments in fast muscles of control (G) or BTS-treated (H) embryos at 72 hpf. I and J. Anti-α-actin antibody staining shows the organization of thin filaments in slow muscles of control (I) or BTS-treated (J) embryos at 60 hpf. K and L. Anti-α-actin antibody staining shows the organization of thin filaments in fast muscles of control (K) or BTS-treated (L) embryos at 72 hpf. Scale bars = 100 µm in A and B, 25 µm in E.

It has been shown that inhibition of myosin ATPase activity by BTS (N-benzyl-p-toluene sulphonamide) blocks thick and thin filament assembly in cultured cells in vitro [24], [25]. Moreover, treating zebrafish embryos with BTS induce paralysis in fish embryos [26]. To determine whether inhibiting myosin function by BTS affects myofibril assembly and muscle contraction in zebrafish embryos *in vivo*, we incubated zebrafish embryos with BTS starting at 12 hpf, a developmental stage correlating with myofibrillogenesis. A clear dose-dependent effect was observed on inhibition of muscle contraction in BTS-treated zebrafish embryos (Table 1). BTS could effectively block muscle contraction at a dose of 20 µM. BTS-treated embryos appeared morphologically normal except the lack of muscle contraction (Fig. 6C). A clear edema and weak cardiac muscle contraction were also detected in BTS treated embryos at 120 hpf (Fig. 6D).

**Table 1:** Dose-dependent effects of BTS on muscle contraction in zebrafish embryos.

BTS	of embryos with normal muscle contraction	# of embryos with weak muscle contraction	# of embryos with no muscle contraction
0.16 µM	53	0	0
0.8 µM	60	6	0
4.0 µM	2	42	0
20 µM	0	0	50
100 µM	0	0	74

Zebrafish embryos were incubated with BTS at different concentrations (0.16 µM, 0.8µM, 4 µM, 20 µM, 100 µM). The BTS treatment started at the 6 somite stage (12 hpf) and continued for 30 hours. Muscle contraction was analyzed in the BTS-treated embryos and characterized in three classes: normal muscle contraction, weak contraction and no contraction.

The myofibril organization of thick and thin filament was analyzed in BTS-treated zebrafish embryos by immunostaining with anti-MyHC (F59), and anti-α-actin antibodies. Unlike the ATG-MO injection, BTS treatment did not significantly reduced the levels of myosin and actin expression in muscle cells (Fig. 6F, J). However, thick and thin filament organization was significantly disrupted in both slow and fast muscles of BTS-treated embryos (Fig. 6F, H, J, L). In contrast, incubation with DMSO, used in making BTS solution, had no effect on thick and thin filament organization (Fig. 6E, G, I, K), confirming that the muscle defects were BTS-specific. Together, these data indicate a critical role for myosin ATPase activity in myosin thick filament assembly and organization. In addition, the myosin-actin interaction is required for thin filament assembly in skeletal muscles.

### 4. Blocking Myosin Function and hsp90α1 Mutation Had Different Effects on Organization of Z- and M-Lines in Skeletal Muscles of Zebrafish Embryos

**Figure 7:**
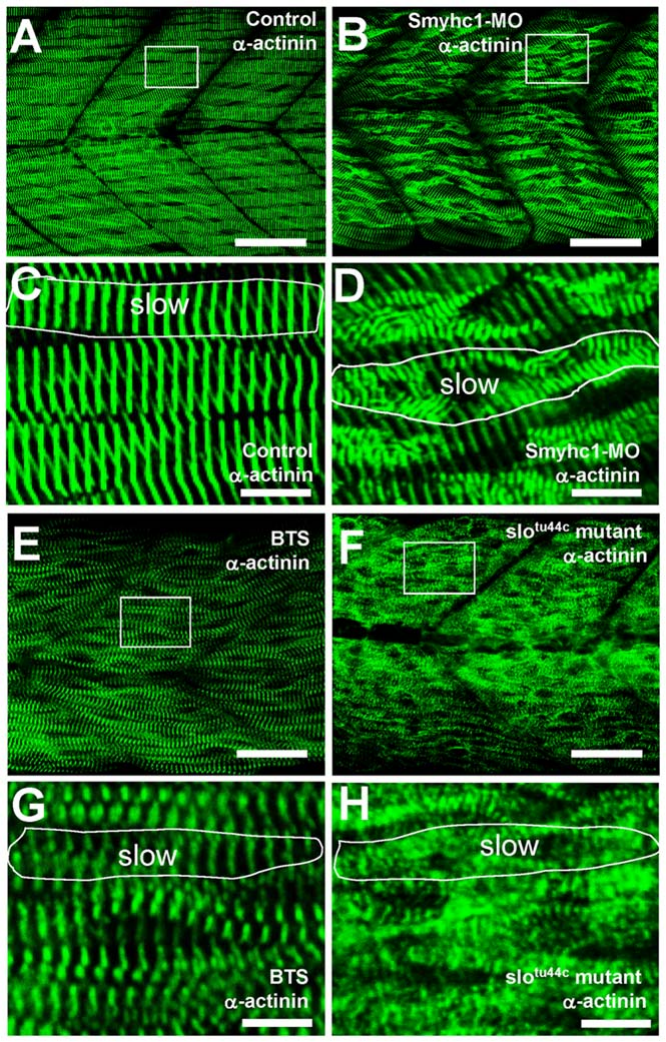
The effect of *smyhc1* knockdown, BTS treatment or *hsp90α1* mutation on Z body formation, and Z-line organization in skeletal muscles of zebrafish embryos. Anti-α-actinin antibody staining shows the Z-disk organization in control (A, C), *smyhc1* knockdown (B, D), BTS-treated (E, G), or *slo^tu44c^* mutant (F, H) embryos at 60 hpf. C, D, G and H are high magnifications of A (control), B (*smyhc1* knockdown), E (BTS treated) and F (*slo^tu44c^* mutant), respectively. Scale bar = 25 µm in A, B; 20 µm in E, F; 4 µm in C, D, G, H.

To determine whether disruption of myosin thick filaments could affect the organization of other sarcomeric structures in skeletal muscles, we analyzed the M- and Z-line structures in *smyhc1* knockdown and BTS-treated zebrafish embryos, and compared them with that from the *hsp90α1* mutation. Immunostaining was performed with anti-myomesin and anti-α-actinin antibodies that specifically label the M- or Z-lines, respectively. Compared with control (Fig. 7A, C), Z-line organization was clearly altered in slow muscles of *smyhc1* knockdown embryos (Fig. 7B, D). Although Z line-like structures were clearly detected in the *smyhc1* knockdown embryos, they failed to align correctly to form the straight Z-line (Fig. 7D). As expected, the Z-line defect was restricted to slow muscles. The Z-lines appeared normal in fast muscles (Fig. 7B, D). However, compared with *smyhc1* knockdown, the Z-line organization appeared less affected in BTS-treated embryos, although the myofibers appeared to be twisted (Fig. 7E, G). In contrast, the Z-line organization was significantly affected in *hsp90α1* mutant embryos (Fig. 7F, H). Very few organized Z-lines could be observed, although Z-bodies could be clearly detected at a higher magnification (Fig. 7H). Together, these data indicate that compared with the loss of myosin function, *hsp90α1* mutation has a strong effect on Z-line organization.

**Figure 8:**
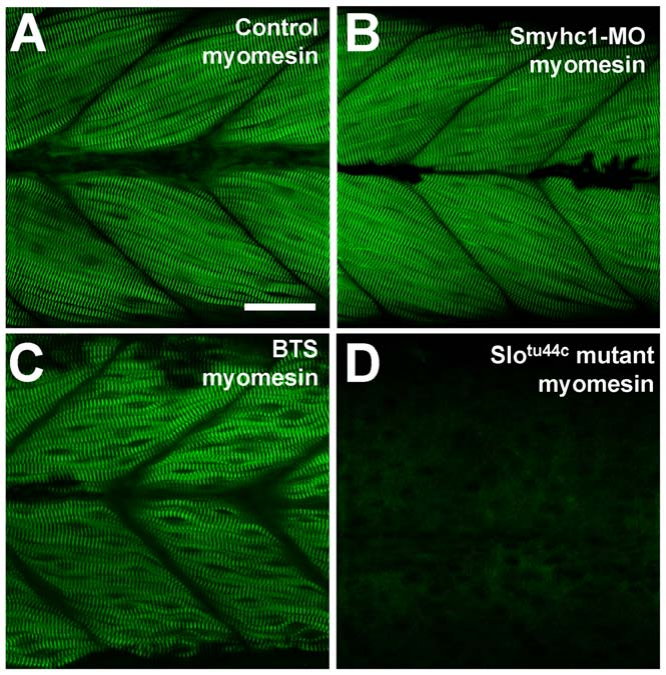
The effect of *smyhc1* knockdown, BTS treatment or *hsp90α1* mutation on M-line organization in skeletal muscles of zebrafish embryos. Anti-myomesin antibody staining shows the M-line organization in control (A), *smyhc1* knockdown (B), BTS-treated (C), or *slo^tu44c^* mutant (D) embryos at 72 hpf. Scale bar = 25 µm in A.

To determine whether M-lines were affected by thick filament disruption in *smyhc1* knockdown, BTS-treated or *slo^tu44c^* mutant embryos, we analyzed M-line organization by immunostaining using an anti-myomesin antibody. The data showed a striking difference in myomesin staining among the three different groups of embryos. Although the organization of M-lines appeared normal in *smyhc1* knockdown and BTS-treated embryos (Fig. 8B, C), the M-line localization of myomesin was completely abolished in *hsp90α1* mutant embryos (Fig. 8D). Very little myomesin staining could be detected in the *hsp90α1* mutant embryos (Fig. 8D), suggesting that the *hsp90a1* mutation could result in a dramatic disruption of M-line organization in skeletal muscles. Collectively, these data indicate that defective thick filament assembly could not account for all myofibril defects in *hsp90a1* mutant embryos, suggesting that Hsp90α1 may play additional roles in the assembly and organization of other sarcomeric structures, such as M-lines in skeletal muscles.

## Discussion

In this study, we analyzed the role of myosin in myofibril assembly and compared it with that of *hsp90α1* mutant in zebrafish embryos. We demonstrated that knockdown of *smyhc1* expression completely disrupted the organization of thick and thin filaments in skeletal muscles of zebrafish embryos. Similarly, inhibiting myosin ATPase with BTS also resulted in disruption of thick and thin filament organization. In contrast, *Hsp90α1* mutation completely disorupted the myofibril organization of all key sarcomeric structures including thick and thin filaments as well as Z- and M-lines. Together, these studies indicate that the myosin chaperone Hsp90α1 is not only important for myosin folding and thick filament assembly, but it may also play additional roles in the organization of other sarcomeric structures such as the M- and Z-lines.

### Smyhc1 Is Required for Thick and Thin Filament Organization in Slow Muscles of Zebrafish Embryos

Zebrafish muscle fibers can be broadly classified into two major types, slow and fast, based on the different contraction speeds, metabolic activities and the expression of MyHC proteins. It has been postulated that zebrafish slow muscles express only a single MyHC isotype [20], [21]. Recent studies indicate that zebrafish slow muscles express at least three types of MyHC isoforms during development [21]. However, Smyhc1 represents the first and predominant MyHC isotype expressed in zebrafish embryonic slow muscles [21]. By using the gene-specific knockdown approach, we demonstrated that Smyhc1 plays a key role in thick filament formation in slow muscles. Knockdown of *smyhc1* expression completely disrupted the organized assembly of thick filaments. It had little effect on the development of fast muscles, consistent with the temporal and spatial patterns of *smyhc1* expression in zebrafish embryos.

The *smyhc1* knockdown phenotype clearly indicates a critical role for myosin thick filaments in the organized assembly of thin filaments in skeletal muscles. This is consistent with previous studies showing that myosin filaments function as an accelerator for actin polymerization *in vitro*, and actin-myosin interaction plays a vital role in myofibril assembly of thin filaments in sarcomeres [27]. This is also consistent with studies in *Caenorhabditis elegans* and *Drosophila melanogaster* that established a clear correlation between *myhc* gene mutation and skeletal muscle disease [28], [29]. In addition, *myhc* mutation disrupts myofibrillar assembly of thick filament in the indirect flight muscle [28].

In contrast to the thick and thin filaments, we showed that knockdown of *smyhc1* expression affected the Z-line organization. However, formation of Z bodies appeared normal, suggesting that the initial formation of Z bodies could occur independently of thick and thin filaments. These data are consistent with our recent studies in zebrafish showing that knockdown of *skNAC* significantly disrupted the assembly of thick and thin filaments, but with a weak effect on the M- and Z-lines [30]. Similar conclusions have been previously reported by Funatsu and colleagues demonstrating that the basic framework of M- and Z-line structures was not significantly affected when both thick and thin filaments were removed *in vitro* [31], [32]. Collectively, these studies support the idea that the basic framework of the sarcomere consisting of the Z-disks and M-bands is established independently prior to the attachment of thick and thin filaments [33], [34], [35].

### BTS Disrupts Thick and Thin Filament Organization in Skeletal Muscles

In this study, we demonstrated that treating zebrafish embryos with BTS, a specific myosin ATPase inhibitor, disrupted myofibril organization in both slow and fast muscles of zebrafish embryos. Results from our *in vivo* studies are consistent with previous findings that BTS suppresses the formation of thick and thin filaments in muscle cells in culture [25], [36], [37]. It should be noted that inhibiting myosin function by BTS exhibited a specific effect on the thick and thin filament organization without much effect on the M- and Z-lines. It is expected that BTS treatment may have a weaker effect compared with the myosin specific knockdown. This is because knockdown blocked the expression of smyhc1, whereas BTS does not block the expression of the myosin protein. Rather, it interferes with its ATPase activity and interaction with a-actin. Because inhibition of myosin function by BTS is a dose-dependent and kinetic process, we expect that some of the myosin protein may not be completely inhibited by BTS and thus resulted in a weaker phenotype compared with smyhc1 knockdown.

Data from our studies provide new evidence that the ATPase activity of the myosin head is required for myofibril assembly. Cheung and collaborators have shown that BTS binds to myosin head domain and inhibits myosin S1 ATPase activity, leading to dissociation of myosin from actin in the presence of ADP [24], [38]. Previous work in *C. elegans* also attested to an important function for the myosin head in thick filament assembly [39]. This study showed that mutations in functionally important domains of the myosin head, including the binding sites for ATP and actin, strongly interfered with assembly of MyHC into thick filaments in body wall muscles [39]. Thus, although the myosin rod is capable of assembly *in vitro* into thick filament-like structures [40], a functional myosin head is required *in vivo* for normal filament formation. However, it should be noted that in contrast to data from *C. elegans* and our present study in zebrafish, a prior report suggests that assembly of thick filaments and myofibrils occurs in the absence of the myosin head in transgenic *D. melanogaster* expressing the headless myosin rod in indirect flight muscles [41]. The cause of the discrepancy is not clear. It could be due to the different model systems used in these studies, or the different approaches used to inactivate or remove the myosin head domain.

### Loss of Myosin Function and hsp90α1 Mutation Have Different Effects on Myofibril Organization

We have demonstrated that loss of myosin function produced a distinct phenotype than *hsp90α1* mutation on myofibril assembly in zebrafish embryos. Although loss of *myosin* or *hsp90α1* function resulted in similar defects in thick and thin filaments, and to certain extent of the Z-line organization, they showed different phenotypes on the M-lines. Loss of myosin function had little effect on the structural organization of M-lines. In contrast, *hsp90α1* mutation completely disrupted myomesin expression and M-line formation in skeletal muscles. This is consistent with the EM characterization of muscle defects in *hsp90α1* mutant embryos [17]. It remains to be determined whether myomesin could represent a potential Hsp90*α*1 client protein. It has been shown that Hsp90 chaperone has numerous client proteins, and plays a key role in their folding, assembly, and activation [42]. Therefore, in addition to myosin, Hsp90α1 may play a vital role in folding and assembly of other sarcomeric proteins, such as myomesin, during myofibrillogenesis in skeletal muscles.

## Materials and Methods

### Zebrafish Maintenance

Mature zebrafish were raised at the zebrafish facility of the Aquaculture Research Center, Center of Marine Biotechnology. The fish were maintained at 28°C with a photoperiod of 14h light and 10h dark, in 8-gallon aquaria supplied with freshwater and aeration. The *slo^tu44c^* mutant zebrafish line was obtained from Tubingen Zebrafish Stock Center [43]. The *slo^tu44c^* mutant carries a nonsense mutation in the *hsp90α1* gene resulting in truncated molecules missing the C-terminal domain, which is important for both homo- and heterodimerization [17], [44].

### Synthesis of Morpholino Antisense Oligos

Morpholino antisense oligos were synthesized by Gene Tools (Corvalis, OR). The *smhyc1* translation blocker (ATG-MO) was targeted to the sequence flanking the ATG start codon. The control-MO was the standard control oligo purchased from Gene Tools.

*smyhc1* ATG-MO: 5′- TCTAAAGTTTTACCCACTGCGGCAA- 3′.

### Microinjection in Zebrafish Embryos

Morpholino antisense oligos were dissolved in 1× Danieau buffer to a final concentration of 0.5 mM or 1 mM. Approximately 1–2 nl (5 ng or 10 ng) was injected into each zebrafish embryo at the 1 or 2 cell stages.

### Analysis of Protein Expression by Western Blot

Chorions were removed from control, or MO-injected embryos (50 embryos for each group) at 24 and 48 hpf. Yolk sacs were removed by gently pipeting embryos through a glass pipet in 1 ml of PBS buffer. The embryos were collected by centrifugation at 5000 rpm for 20 seconds. The pellet of embryos was dissolved in 150 µl of 2× SDS loading buffer (3 µl for each embryo), and homogenized with a 21# needle/syringe. 2 µl of PMSF was added to reduce the bubbles. The sample was boiled for 3 min at 100C. 20 µl of protein sample was analyzed on a 7.5% SDS PAGE. The proteins were transferred onto an Immobilon-P membrane (Millipore) and immunostaining was carried out using anti-MHC (F59; DSHB), and anti-γ-tubulin (T6557; Sigma) antibodies.

### Whole Mount In Situ Hybridization

The cDNA of zebrafish slow-specific troponin C (stnnc; NM_001002085) was cloned from zebrafish embryos (24 hpf) by RT-PCR using the First-strand cDNA synthesis kit and followed by an advantage-2 DNA polymerase (Clontech). Stnnc-p1 and stnnc-p2 primers were used in the PCR reaction [stnnc-p1, 5′-aatgatgtatataaagcagcggtg-3′; stnnc-p2, 5′-tttactcgacacccttcataaagt-3′]. The PCR products were cloned into pGEM-T easy vector (Promega) and sequenced. The resulting plasmids were named *pGEM-stnnc*.

Whole mount in situ hybridization was carried out using digoxigenin-labeled antisense probe in *smyhc1* knockdown and control embryos at 24 hpf. Plasmid *pGEM-stnnc* was digested with Spe I and transcribed with T7 RNA polymerase to synthesize the digoxigenin-labled antisense RNA probe.

### Construction of ef1α:smyhc1 Gene Construct and Rescue Experiment

To generate a DNA construct that can be used to rescue the *smyhc1* knockdown phenotype in myofibers of zebrafish embryos, we constructed the *ef1α:smyhc1* plasmid that contains the zebrafish elongation factor 1α (*ef1α*) promoter and the full length *smyhc1* cDNA coding sequence. Briefly the full length *smyhc1* cDNA of was amplified by RT-PCR using zebrafish embryos of 24 hpf. The PCR was carried out using Pfu DNA polymerase (Stratagene) with smyhc1-ATG and smyhc1-stop primers. The PCR product was phosphorylated by T4 Kinase (Promega) and cloned into the Stu I site of pCS2 vector to generate the *cmv:smyhc1* plasmid. The CMV promoter in the *cmv:smyhc1* plasmid was removed by Sal I and BamH I digestion and replaced by the ef1α promoter from pT2AL200R150G plasmid [45]. The final construct was named *ef1α:smyhc1*.

smyhc1-ATG primer: 5′ –accatgggtgacgccgttatggca- 3′

smyhc1-stop primer: 5′ –gattacccttcatcatgtcctttcttg- 3′

The rescue experiment was performed by co-injecting *smyhc1* ATG-MO (5ng) with 50 pg of *ef1α:smyhc1* plasmid DNA. Injection of *ef1α:smyhc1* plasmid alone was used as control to show the expression of the construct by whole mount antibody staining with F59 antibody at 12 and 22 hpf.

### BTS (N-benzyl-p-toluene sulphonamide) Treatment

BTS (S949760, Sigma) stock solution (50 mM) was prepared in dimethylsulfoxide (DMSO). For dose-dependent analysis, zebrafish embryos were incubated with BTS in fish water containing different concentrations of BTS (0.16 µM, 0.8 µM, 4 µM, 20 µM, 100 µM). The treatment started at the 6 somite stage (12 hpf) and stopped 30 hours after the treatment.

For analysis of BTS-induced myofibril defects, zebrafish embryos (100 each dish) were incubated with 50 µM of BTS. The treatment started at the 6 somite stage (12 hpf) and terminated at 36, 60 and 72 hpf by fixing in 4% paraformaldehyde for use with antibody staining.

### Whole-Mount Immunostaining

Immunostaining was carried out using whole-mount zebrafish embryos (1–3 days post-fertilization) as previously described [15], [46]. Briefly, zebrafish embryos were fixed in 4% paraformaldehyde (in PBS) for 1 hour at room temperature. The fixed embryos were washed for 15 minutes 3 times in PBST. Three day old embryos were digested in 1 mg/ml collagenase for 75 minutes. Immunostaining was performed with the following primary antibodies: anti-α-actinin (clone EA-53, #A7811, Sigma), anti-MyHC for slow muscles (F59, DSHB), anti-myosin light chain for fast muscles (F310, DSHB), anti-MyHC (MF-20, DSHB), anti-myomesin (mMaC myomesin B4, DSHB), and anti-α−actin (Ac1-20.4.2, Progen). Secondary antibodies were FITC conjugates (Sigma).

### Statistical Evaluation

All these studies have been conducted in triplicate. Approximately 100 embryos were analyzed for each injection and treatment per experiment. Moe than seven hundred of embryos have been analyzed during the course of this study. Among those analyzed embryos, over 95% of the *smyhc1* knockdown embryos and 100% of the BTS (20 µM) treated embryos showed the muscle defects, indicating that the effects from the knockdown and BTS treatment were statistically significant.
